# Hemianopsia

**Published:** 1903-03-28

**Authors:** 


### HEMIANOPSIA.

In the *New York Medical News* of February 28th, Dr. Edward Jackson gives a detailed report of two cases of hemianopsia which occurred as a result of definite, localised cerebral injuries. In one of these patients the half-blindness was complete, in the other it took the form of a partial right hemianopsia, affecting the right lower quadrants of the fields of vision only. Dealing with the condition in its general aspects Dr. Jackson remarks that it is one which is particularly liable to be overlooked, and for this reason, that unless the patient is examined with special reference to such a defect and the field of vision is carefully investigated, a diagnosis cannot be made. The patient's statements are occasionally very suggestive if the loss of vision affects the immediate neighbourhood of the fixation point; in such a case the complaint may be that only half of an object looked at is visible. More often advice is sought for blindness in one eye; this is explained by the difference in size between the nasal and temporal portions of the field of vision, the latter being much the larger, and consequently the defect is more noticeable in the eye whose temporal portion of the visual field is curtailed. Dr. Jackson has noted that the division line between the two portions of the visual field is not a straight one, but that the seeing field encroaches upon the blind part at the region of the fixation point. Besides this there are other irregularities in the line of demarcation, and these irregularities correspond in the two eyes. The first case described is that of a labourer, aged 48 years, who four years previously fell on to his head and was stunned. No serious symptoms followed immediately.

Subsequently impairment of vision developed and examination revealed a fracture of the occipital bone. Operation showed fragments of the inner table driven into the cortex of the occipital lobe. Beginning 2 inches above the occipital protuberance, half an inch to the right of the median line, was a depression which extended to the right and about 15° upwards for 2 inches, and which was about half an inch wide. The fields of vision showed left lateral hemianopsia. The pupils reacted normally to light thrown on either half of the retina. The other case was in a man of 50 years who, 30 years previously, had received a gunshot wound of the occipital region. He stated that he was entirely blind for a few days after the injury, and subsequently saw through a haze. He still complained of attacks of hazy vision preceded by vertigo and followed by headache. On examination a depression of the skull was found 1 inch in diameter, its centre $\frac{1}{2}$ inch to the right of the median line and $1\frac{1}{2}$ inch above the occipital protuberance. This marked the wound of entrance. From this a groove $\frac{1}{2}$ inch wide extended down and to the left to a point $1\frac{1}{4}$ inch to the left of the median line and $\frac{1}{2}$ inch above the occipital protuberance. This was the point of exit. The blindness was confined to the right lower quadrants of the field of vision. The reactions of the pupils were normal. In case (1) the brain lesion progressed after the injury as shown by the history. In both cases the upper lip of the calcarine fissure must have been involved, which portion of the cortex is thought by some to govern the upper parts of the retina, and consequently the lower quadrants of the field of vision. Dr. Jackson thinks that both his cases lend support to that view.

